# Crystal structures of human immune protein FIBCD1 suggest an extended binding site compatible with recognition of pathogen-associated carbohydrate motifs

**DOI:** 10.1016/j.jbc.2023.105552

**Published:** 2023-12-10

**Authors:** Harry M. Williams, Jesper B. Moeller, Ian Burns, Anders Schlosser, Grith L. Sorensen, Trevor J. Greenhough, Uffe Holmskov, Annette K. Shrive

**Affiliations:** 1 School of Life Sciences, Keele University, Staffordshire, United Kingdom; 2 Cancer and Inflammation Research Unit, Department of Molecular Medicine, University of Southern Denmark, Odense, Denmark; 3 Danish Institute for Advanced Study, University of Southern Denmark, Odense, Denmark

**Keywords:** FIBCD1, crystal structure, binding studies, chitin, innate immunity, ficolins, host–pathogen interaction, structural biology

## Abstract

Fibrinogen C domain-containing protein 1 (FIBCD1) is an immune protein proposed to be involved in host recognition of chitin on the surface of pathogens. As FIBCD1 readily binds acetylated molecules, we have determined the high-resolution crystal structures of a recombinant fragment of the FIBCD1 C-terminal domain complexed with small N-acetyl-containing ligands to determine the mode of recognition. All ligands bind at the conserved N-acetyl-binding site (S1) with galactose and glucose-derived ligands rotated 180° relative to each other. One subunit of a native structure derived from protein expressed in mammalian cells binds glycosylation from a neighboring subunit, in an extended binding site. Across the various structures, the primary S1 binding pocket is occupied by *N*-acetyl-containing ligands or acetate, with *N*-acetyl, acetate, or sulfate ion in an adjacent pocket S1(2). Inhibition binding studies of *N*-acetylglucosamine oligomers, (GlcNAc)_n_, n = 1, 2, 3, 5, 11, *via* ELISA along with microscale thermophoresis affinity assays indicate a strong preference of FIBCD1 for longer *N*-acetylchitooligosaccharides. Binding studies of mutant H396A, located beyond the S1(2) site, showed no significant difference from wildtype, but K381L, within the S1(2) pocket, blocked binding to the model ligand acetylated bovine serum albumin, suggesting that S1(2) may have functional importance in ligand binding. The binding studies, alongside structural definition of diverse *N*-acetyl monosaccharide binding in the primary S1 pocket and of additional, adjacent binding pockets, able to accommodate both carbohydrate and sulfate functional groups, suggest a versatility in FIBCD1 to recognize chitin oligomers and other pathogen-associated carbohydrate motifs across an extended surface.

Fibrinogen C domain-containing protein 1 (FIBCD1) is a type II transmembrane receptor in the fibrinogen-like recognition domain (FReD) superfamily and the first human receptor identified that binds specifically to chitin (1). FIBCD1 is encoded at 9q34.12-13 in humans, adjacent to the genes that encode the homologous immune proteins ficolin-1 (*FCN1*) and ficolin-2 (*FCN2*). Human FIBCD1 is expressed in epithelial cells derived from all three germ layers with epithelial cells throughout the gastrointestinal tract and the respiratory system demonstrating notably high levels of expression (2). Chitin, the second most abundant biopolymer after cellulose and a major component of the fungal cell wall, is known to modulate the human immune response. Following successful ligation, FIBCD1 mediates the endocytosis of its bound ligand, directing acetylated components for endosomal degradation (1). Recently, it was demonstrated that FIBCD1 directly bound to intestinal-derived fungi and that *in vivo* expression of FIBCD1 led to significantly reduced fungal colonization as well as a reduction in fungal-mediated intestinal inflammation (3). In the lungs, data indicate that FIBCD1 has a detrimental role in the development and progression of invasive pulmonary aspergillosis (4) where cytokine and chemokine expression associated with chitin-containing *Aspergillus fumigatus* are modulated by FIBCD1 expression (5). Further evidence demonstrating that FIBCD1 is a receptor for chondroitin sulfate proteoglycans of the brain extracellular matrix has also been reported (6), while early data suggest a protective role for soluble FIBCD1 as a myokine *in vivo* in a model for cancer-induced myofiber atrophy (7).

FIBCD1 forms tetramers in the plasma membrane, each monomer consisting of a small cytoplasmic domain, a short trans-membrane helix, and a large ectodomain composed of a coiled-coil region, a polycationic region, and the FReD (8). FIBCD1-FReD demonstrates homology to the fibrinogen domains of ficolin-1, ficolin-2, and ficolin-3 with protein residues involved in *N*-acetyl coordination and metal binding largely conserved across the ficolins and FIBCD1 (9). Similar to the homotrimeric ficolins, it is thought that FIBCD1 may use oligomerization to establish the appropriate spatial arrangement of its FReD domains allowing it to interact with microbial cell surface ligand structures while leaving endogenous acetylated structures unbound due to their alternative spacing (10).

FIBCD1 binds readily to acetylated molecules, such as fungal chitin and sialic acid, in a calcium-dependent manner with ligands coordinated *via* a hydrophobic and aromatic pocket (8), designated the S1 site (named according to the corresponding site in the ficolin proteins (11)). Crystal structures of a recombinant glycosylated fragment of FIBCD1-FReD have been reported both without bound ligand and bound to *N*-acetyl-D-mannosamine (ManNAc) (10). The native structure, in which the N-linked glycosylation from one subunit is found bound in the ligand-binding site of a neighboring subunit *via* a crystal contact, together with the ManNAc-bound structure, has provided key insights into ligand coordination and selectivity revealing that ligands are bound at the S1 site principally through their *N*-acetyl group. Two tyrosine residues flanking the S1 site, Tyr405 and Tyr431, are also thought to stabilize protein–ligand interactions (10). While other classical pathogen-associated molecule patterns, such as lipopolysaccharide, lipoteichoic acid, or peptidoglycan are not bound by FIBCD1 (1), recognition of polysaccharide structures other than chitin in the structural skeleton of the *A. fumigatus* cell wall may occur and may utilize a binding site different from, or perhaps extending beyond, the S1 site on FIBCD1 (12).

Ficolin-1 and the homologous tachylectin 5A (TL5A) also bind acetylated ligands *via* the S1 site with each coordinating ligands in a similar manner to FIBCD1 (13, 14). In ficolin-2, changes to a number of residues at the S1 site render it inactive and, instead, ligands are coordinated at alternative sites (designated S2, S3, and S4), which in ficolin-2 form an extended binding surface upon which a range of carbohydrate and noncarbohydrate ligands are bound (11, 15, 16). Whether these additional sites (S2-4) are functional in FIBCD1 remains to be seen, although both published structures of FIBCD1-FReD show sulfate bound at the S3 site, suggesting FIBCD1 may be able to coordinate sulfated ligands *via* this site (10). NMR and modeling studies of chitin-binding hevein domains with bound *N*-acetylglucosamine (GlcNAc) oligosaccharides ((GlcNAc)_n_, n = 1–6) have revealed an extended chitin-binding site where nonterminal sugars of the longer fragments may bind at the primary acetyl site (17, 18). Recent atomistic simulations for (GlcNAc)_3_ suggest the presence of three binding pockets in the extended hevein site, one for each of the three sugars, with the nonreducing terminal sugar located in the primary acetyl-binding site (19).

The aim of this study is to build on and further develop our current understanding of how FIBCD1, *via* the FReD, detects invading pathogens through the recognition of known acetylated ligands. This has been achieved by combining studies on the relative binding affinities of FIBCD1-FReD for *N*-acetylchitooligosaccharides with the determination of a series of high-resolution crystal structures of FIBCD1-FReD bound to small *N*-acetylated ligands known to be recognized by FIBCD1 (1).

## Results

### Crystal structures

The high-resolution crystal structures of a recombinant fragment of FIBCD1-FReD in complex with *N*-acetylalanine (1.94 Å), GlcNAc (1.84 Å), GalNAc-4S (1.97 Å), Neu5Ac (1.93 Å), and *N*,*N*′-diacetylchitobiose ((GlcNAc)_2_, 1.85 Å) have been determined. Additionally, data from a Chinese hamster ovary (CHO)-derived protein crystal soaked with a short chondroitin sulfate A/C (CSA/C) mixed oligomer failed to reveal bound ligand; however, processing of these data has provided a native structure with further definition of the N-linked glycosylation at Asn340. The unit cells of the structures reported here contain two independent tetramers, one composed of subunits A, the other of subunits B, each with 4-fold molecular symmetry. With the exception of the *N*-acetylalanine ligand-bound structure, in which the ligand is found in both subunits, bound ligand is only observed in subunit A with the electron density in the subunit B *N*-acetyl site S1 corresponding to that of an acetate ion coordinated by Cys414, His415, and in some cases Tyr431 OH, rather than any soaked ligands (Fig. S1). The subunit A S1 site in the native structure is similarly occupied by an acetate ion as reported (10). There is also electron density present in the ficolin-associated S2 site in several of the structures with sufficient density in some cases to model an acetate ion.

In all the crystal structures, a crystal contact places the subunit A Asn340 glycosylation proximal to the subunit B S1 binding site interface, interacting across an extended subunit B surface, which incorporates S1 for the native structure (10). The protein used for the ligand-bound structures was expressed in insect cells with the glycosylation at Asn340 being characteristically insect with difucosylation, by both α1,3- and α1,6-fucose, of the core reducing-terminal GlcNAc (20). A glycosylation pattern that more closely aligns to human glycosylation is seen in the new native structure derived from protein expressed in mammalian CHO cells.

### Ligand-bound structures

**Figure 1:**
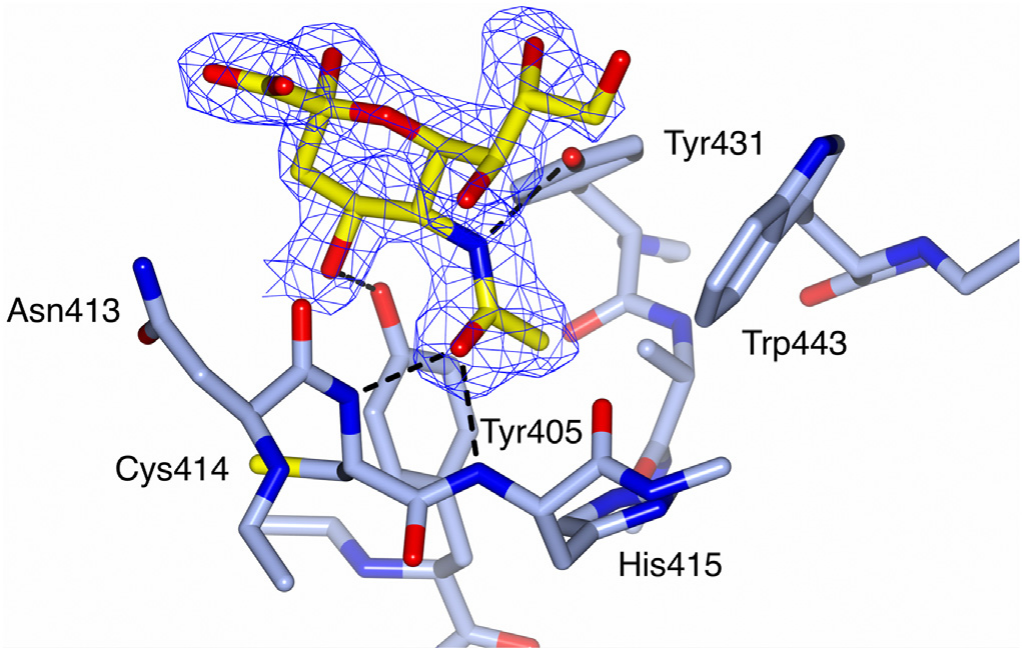
**Electron density for the Neu5Ac ligand bound to FIBCD1-FReD.** Bound Neu5Ac ligand (*yellow*) shown in the subunit A S1(1) ligand-binding site (*pale blue*). *N*-acetyl interactions of ligand with protein are shown by *dashed lines*. The 2mFo−DFc electron density map (*blue*) is clipped to the bound Neu5Ac ligand and contoured at 1σ.

While the quality of the electron density for the bound ligands varies between the structures, all the ligands can be clearly fitted, except for a second GlcNAc in the (GlcNAc)_2_-bound structure, which is not visible in the electron density (see Fig. S2). Ligand coordination is achieved in the subunit A primary *N*-acetyl-binding site S1 (designated S1(1) here) by the ligand *N*-acetyl group, which is coordinated by the backbone nitrogen atoms of Cys414 (2.61–2.76 Å) and His415 (2.82–3.05 Å), and the side-chain hydroxyl of Tyr431 (2.77–3.00 Å) (Fig. 1 and Table 1). These interactions, which are supplemented by extensive water-mediated contacts and in some cases by an interaction with the Tyr405 OH group, restrict the ligand *N*-acetyl group to a hydrophobic and aromatic pocket.

**Table 1:** Protein to ligand, ion, and glycan binding distances (Å) in the S1(1), S1(2), and S1(3) binding pockets

Binding Pocket	Atom1	Atom2											
		Native		AlaNAc		GlcNAc		GalNAc-4S		Neu5Ac		(GlcNAc)_2_	
Subunit A													
**S1(1)**	Cys414 N	ACY O	2.86	OT	2.72	O7	2.64	O7	2.61	O10	2.76	O7	2.75
	His415 N	ACY O	2.98	OT	2.96	O7	3.05	O7	2.86	O10	2.82	O7	2.82
	His415 O	ACY O	3.38										
	Tyr431 OH	ACY OXT	3.14	N	3.00	N2	2.77	N	2.86	N5	2.93	N2	2.96
	Tyr405 OH									O4	2.65	O1	3.07
**S1(2)**	Asn413 O	SO4 O4	3.39	ACY O	3.37			OSC	2.69				
	Asn413 N	SO4 O4	2.97	ACY O	3.00								
Subunit B													
**S1(1)**	Cys414 N	NAG1 O7	2.67	OT	2.84	ACY O	2.61	ACY O	2.72	ACY O	2.81	ACY O	2.65
	His415 N	NAG1 O7	2.92	OT	2.79	ACY O	2.94	ACY O	3.00	ACY O	3.04	ACY O	2.86
	His415 O	NAG1 O3	3.04										
	Tyr431 OH	NAG1 N2	3.01	N	3.26			ACY OX	3.06	ACY OX	3.25		
	Tyr405 OH			O	2.78								
**S1(2)**	Asn413 O	NAG2 O7	3.37	OXT	3.00								
	Asn413 N	NAG2 O7	2.98										
**S1(3)**	His396 ND1	Man O2	3.04	SO4 O1	2.59	SO4 O2	2.55	SO4 O1	2.76	S04 O1	2.64	SO4 O1	2.61
	Arg412 NH1	Man O2	3.28	SO4 O1	2.75	SO4 O2	3.06	SO4 O1	2.81	S04 O1	2.99	SO4 O1	2.78
	Tyr431 OH	Asn O A340	3.20										
	Asn340 (sub A) ND2			OXT	2.78								

Acetate ion is indicated as ACY, sulfate ion as SO4, and the N-linked glycan as NAG1-NAG2-Man. Atoms in the bound ligands are underlined.

**Figure 2:**
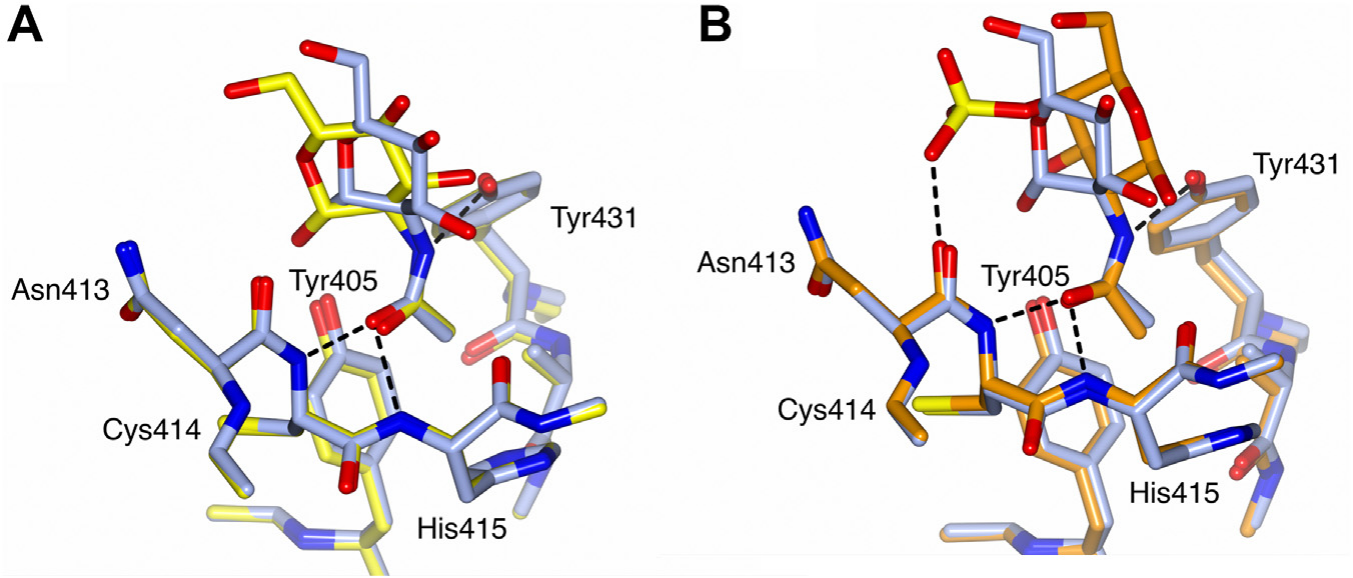
**GlcNAc, (GlcNAc)**_**2**_**, and GalNAc-4S binding in the FIBCD1-FReD subunit A S1(1) pocket showing key amino acids and interactions between bound ligand and protein.** Overlay of GlcNAc bound structure (*pale blue*) with (*A*) (GlcNAc)_2_ bound structure (*yellow*) showing amino acid interactions for GlcNAc. (GlcNAc)_2_ interactions are the same except for an additional interaction of O1 with Tyr405. *B*, GalNAc-4S structure (*orange*) showing amino acid interactions with GalNAc-4S. Overlays were generated by a least-squares fit of subunit A main chains.

The **GlcNAc and (GlcNAc)**_**2**_ structures both show a GlcNAc bound in the subunit A primary *N*-acetyl site S1(1) (Fig. 2*A*). A difference in orientation of the bound GlcNAc in the two structures results in an additional interaction (3.07 Å) between the Tyr405 side-chain hydroxyl and the O1′ hydroxyl of the bound GlcNAc in the (GlcNAc)_2_ structure. There is no electron density for a second GlcNAc of (GlcNAc)_2_, suggesting that the second GlcNAc is mobile within the structure. It is not possible to distinguish whether the GlcNAc visible in the electron density is reducing or nonreducing. In the **GalNAc-4S and Neu5Ac** ligand-bound structures the galactopyranose ring is rotated about the C-N bond by approximately 180° compared with the glucopyranose rings in the GlcNAc and (GlcNAc)_2_ ligand-bound structures (Figs. 1 and 2). In the Neu5Ac structure (Fig. 1) this rotation enables an interaction between the Tyr405 side-chain hydroxyl and the Neu5Ac O4′ hydroxyl (2.65 Å). While the electron density for the GalNAc-4S ligand is sufficiently well defined to place and model the ligand, it is not as well defined as for the other ligand-bound structures. This may be due to the crystal wells having been initially soaked with GalNAc, followed at a later date by soaking with GalNAc-4S. The GalNAc-4S sulfate group is positioned sufficiently close to the FReD such that there is a weak van der Waals clash between a sulfate oxygen and the Asn413 main-chain carbonyl (2.69 Å) (Fig. 2*B*). Repositioning the sulfate oxygens to alleviate this clash was attempted, but refinement consistently returned the sulfate group to this position. A similar GalNAc-4S–main chain carbonyl van der Waals interaction (O-O 2.8 Å) is seen in at least one other crystal structure (Protein Data Bank [PDB] ID 7JGH, ref (21)). The ***N*-acetylalanine** ligand is seen in the primary *N*-acetyl site S1(1) of both subunits (Table 1 and Fig. S3). In subunit B this ligand also makes further interactions in addition to those with the primary *N*-acetyl site, the ligand carboxylate group interacting with the Tyr405 side-chain hydroxyl (2.78 Å), and the subunit A Asn340 side chain (Fig. S3*B*), due to the close proximity of the Asn340 N-linked glycan from subunit A.

The N-linked glycosylation derived from expression in insect cells is variably defined with the subunit A glycan interacting with subunit B, across the crystal contact *via* a surface in proximity to but not including the primary *N*-acetyl-binding site (see Fig. S1). The first GlcNAc residue and an α-1,3-linked L-fucose residue of the glycan are seen in the electron density for all the ligand-bound structures, with the fucose O2 and O3 both interacting with the subunit B Glu398 main-chain nitrogen (2.89–3.25 Å) with additional interactions between O2 and the Asn413 side chain and between O3 and the His396 main-chain carbonyl. A second GlcNAc residue in the glycosylation chain, also interacting with the Asn413 side chain and the His396 main-chain carbonyl, is defined in the Neu5Ac and GlcNAc-bound structures. Additionally, an L-fucose α-1,6-linked to the Asn340-linked GlcNAc is also seen in the electron density for the GlcNAc-bound structure (Fig. S1), and there is evidence of this fucose in the Neu5Ac and (GlcNAc)_2_-bound structures, although the density is not sufficiently defined to allow fitting with confidence.

### Extended ligand-binding site

**Figure 3:**
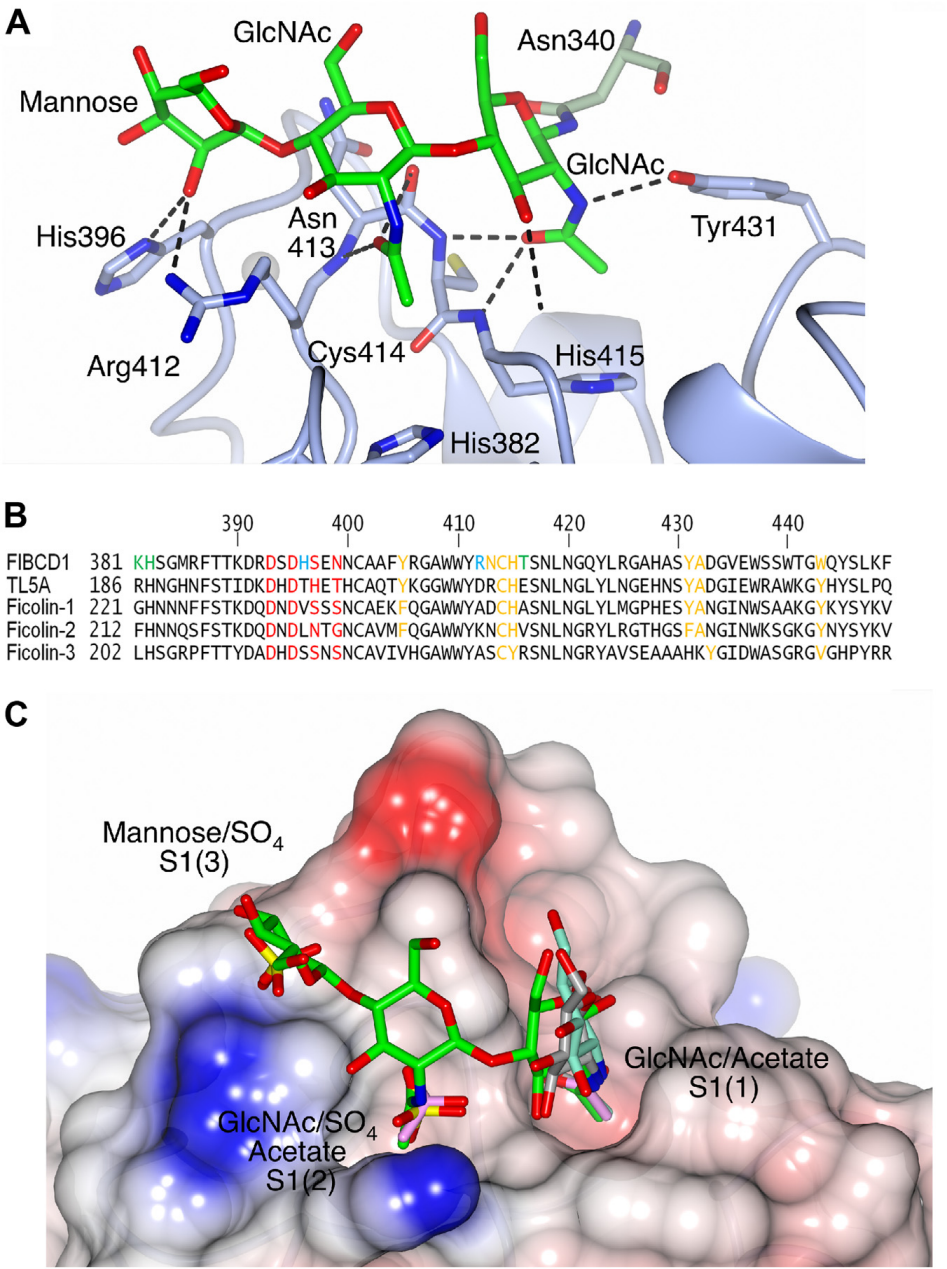
**The S1 binding site on FIBCD1.***A*, native glycan bound in the FIBCD1 subunit B S1 site. The Asn340-linked glycan is shown as a *stick model* (*green*) binding to the subunit B S1 ligand-binding site (*blue*). *B*, sequence alignment in the S1 ligand-binding region in FIBCD1 and homologous proteins TL5A, Ficolin-1, Ficolin-2, and Ficolin-3. Sequence numbers for each protein domain are indicated. The primary *N*-acetyl S1(1) binding pocket residues are highlighted in orange and calcium coordinating residues in red. Additional residues involved in the neighboring pocket (S1(2)) in FIBCD1 are highlighted in *green* and residues His396 and Arg412, involved in forming S1(3) on FIBCD1-FReD, are highlighted in *cyan*. *C*, FIBCD1 surface view showing the native glycan (*green*) bound in subunit B with the N-linked GlcNAc in the primary S1(1) conserved pocket; the second GlcNAc in the neighboring pocket, designated S1(2); and mannose in the designated S1(3) pocket. Overlaid (least-squares fit of main-chain residues of each structure) in the S1(1) pocket are GlcNAc from the GlcNAc-bound structure (*gray*) and from the (GlcNAc)_2_ ligand-bound structure (*cyan*) and acetate (*pink*, present in the subunit A S1(1) of native and subunit B of the GlcNAc, GlcNAc2, Neu5Ac, and GalNAc4S structures). The sulfate ions in S1(2) of subunit A of the native structure and in S1(3) of subunit B of all the ligand bound structures are also shown along with the acetate ion in the subunit B S1(2) pocket of the *N*-acetylalanine structure.

In the CHO-derived native structure, refined to 2 Å, there is electron density present for the subunit A N-linked GlcNAc in the subunit B primary *N*-acetyl-binding site S1(1) (Fig. 3), along with sufficient density to allow the fitting of a second β-1,4-linked GlcNAc and a mannose residue. The core reducing terminal GlcNAc is bound in S1(1) *via* Cys414, His415, and Tyr431, as described by Shrive *et al.* (Ref (10)) (Fig. 3). The *N*-acetyl oxygen of the second β-1,4-linked GlcNAc of the glycan is placed in a secondary acetyl-binding pocket, named here as S1(2), in a cleft neighboring S1(1), forming contacts with the backbone nitrogen atom of Asn413 (2.98 Å) along with the Asn413 main-chain carbonyl (3.37 Å). The side chain of His382 forms the base of this pocket. Coordination of the glycan mannose residue by the side chains of His396 (Man O2-His396 ND1 3.04 Å) and Arg412 (Man O2-Arg412 NH1 3.28 Å) results in further extension of the glycan-binding site, named S1(3) here for clarity (Fig. 3 and Table 1).

In subunit B of each ligand-bound crystal structure, electron density proximal to the binding pocket S1(3) observed for the glycan mannose in the CHO-derived native structure has been modeled as a sulfate ion. In common with the glycan mannose in the native structure (Fig. 3*A*), this sulfate is coordinated by the side chains of His396 (ND) and Arg412 (NH) at 2.55 to 3.06 A (Figs. 3*C* and S1). Sulfate ions have also been modeled in the crystal structures at two sites previously identified by Shrive *et al.* (Ref (10)) (Fig. S4). The first site corresponds to the ligand-binding site in ficolin-2 known as the S3 site (11). This sulfate, which is found in both subunits A and B of all the crystal structures reported here, is coordinated by the Arg297 side chain, the backbone nitrogen of Gly298, and in subunit B the Lys390 (NZ) side chain. The second site, which is only observed in subunit A of the native structure, is located in the secondary acetyl-binding pocket S1(2) (Fig. 3*C*). This S1(2) sulfate interacts with protein main-chain Asn413 in a similar manner to the GlcNAc of the native glycan in S1(2) (Table 1). An acetate ion in S1(2) of subunit A of the *N*-acetylalanine-bound structure also interacts with Asn413 in a similar manner (Fig. S3*A*).

### Binding studies

**Figure 4:**
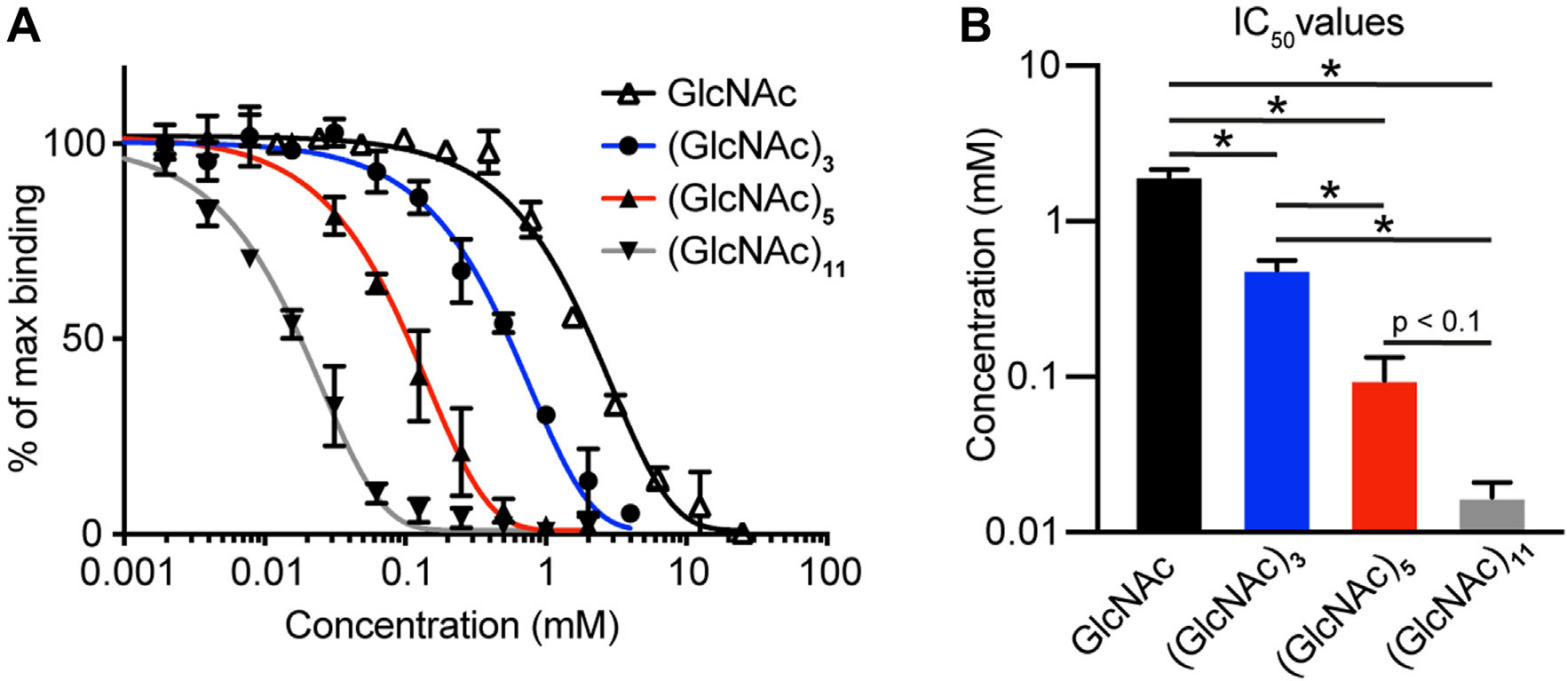
**Inhibition of FIBCD1-FReD binding to acetylated bovine serum albumin by *N*-acetylchitooligosaccharides.***A*, representative ELISA-based setup demonstrating acetyl group–specific inhibition of binding between FIBCD1-FReD and AcBSA by the tested *N*-acetylchitooligosaccharides. *B*, statistical comparison of the tested *N*-acetylchitooligosaccharides and their ability to inhibit binding between FIBCD1-FReD and AcBSA at half maximal inhibitory concentration (IC50). Results shown in *A* and *B* are combined data from three to four independently performed experiments. Statistics: Data are presented as mean ± SD. ∗*p* < 0.05 by one-way analysis of variance (ANOVA) with *post hoc* Tukey test.

**Figure 5:**
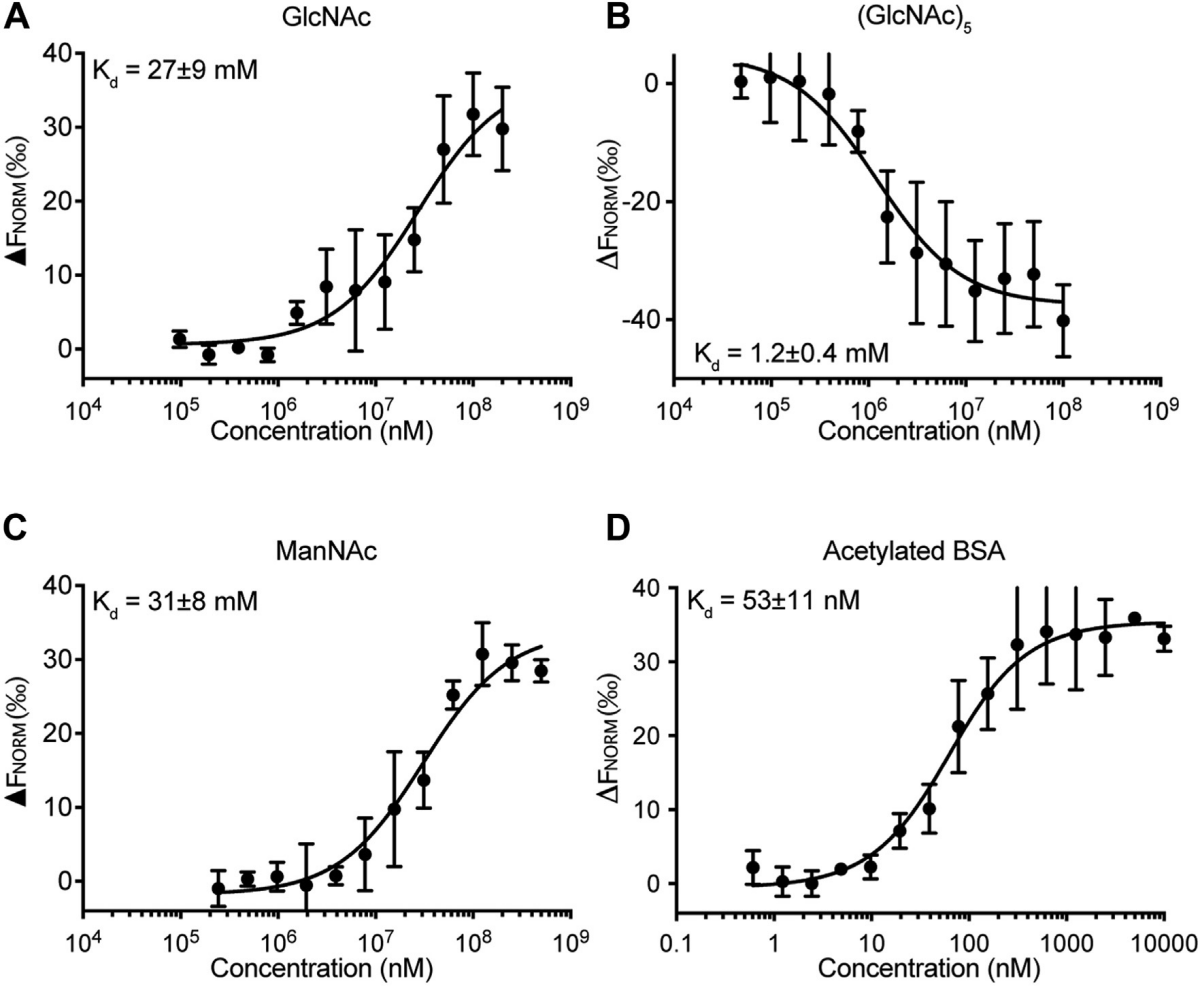
**Analysis of FIBCD1-FReD interactions with acetylated molecules by microscale thermophoresis.** Representative microscale thermophoresis binding curves of recombinant FIBCD1-FReD with **(***A*) *N*-acetylated glucosamine (GlcNAc), (*B*) *N*-acetylchitooligosaccharide (GlcNAc)_5_, (*C*) *N*-acetylated mannosamine (ManNAc), and (*D*) acetylated BSA (AcBSA). The affinity of FIBCD1-FreD to AcBSA (53 ± 11 nM), GlcNAc (27 ± 9 mM), (GlcNAc)_5_ (1.2 ± 0.4 mM), and ManNAc (31 ± 8 mM) was determined employing the Thermophoresis + T-jump signal for data analysis. Results shown in A to D are combined data from three to four independently performed measurements. Data are presented as mean ± SD.

The binding of FIBCD1-FReD with GlcNAc, (GlcNAc)_3_, and longer *N*-acetylchitooligosaccharides of defined length ((GlcNAc)_5_ and (GlcNAc)_11_) was investigated by ELISA. The relative affinity for these carbohydrates was assessed by inhibiting the binding of FIBCD1 to immobilized acetylated bovine serum albumin (AcBSA) in microtiter plates. The use of AcBSA or other poly-acetylated model ligands has previously been shown to provide a stable and consistent immobilized surface in ELISA-based setups for the characterization of acetyl-group-specific binding characteristics (1, 13). The assays revealed that the longest *N*-acetylchitooligosaccharide tested, (GlcNAc)_11_, inhibited the binding to AcBSA by 50% (IC50) at 0.02 ± 0.003 mM, far more effectively than (GlcNAc)_5_ (0.1 ± 0.03 mM), (GlcNAc)_3_ (0.5 ± 0.06 mM), or GlcNAc (2.0 ± 0.22 mM) (Fig. 4). The ligand-binding properties of FIBCD1-FReD were further evaluated using microscale thermophoresis (MST) measurements. The MST analyses revealed a 23-fold difference in the affinity for GlcNAc (K_d_ = 27 ± 9 mM) and (GlcNAc)_5_ (K_d_ = 1.2 ± 0.4 mM), GlcNAc exhibiting a similar affinity to ManNAc (K_d_ = 31 ± 8 mM) (Fig. 5, *A*–*C*). Moreover, the MST experiments revealed a significantly higher affinity toward the poly-acetylated model ligand AcBSA (K_d_ =53 ± 11 nM) compared with all other tested ligands (Fig. 5*D*). Combined, these data show that, as the length of the *N*-acetylchitooligosaccharide increases (increased number of acetyl groups), there is an increase in affinity for binding by FIBCD1-FReD.

**Figure 6:**
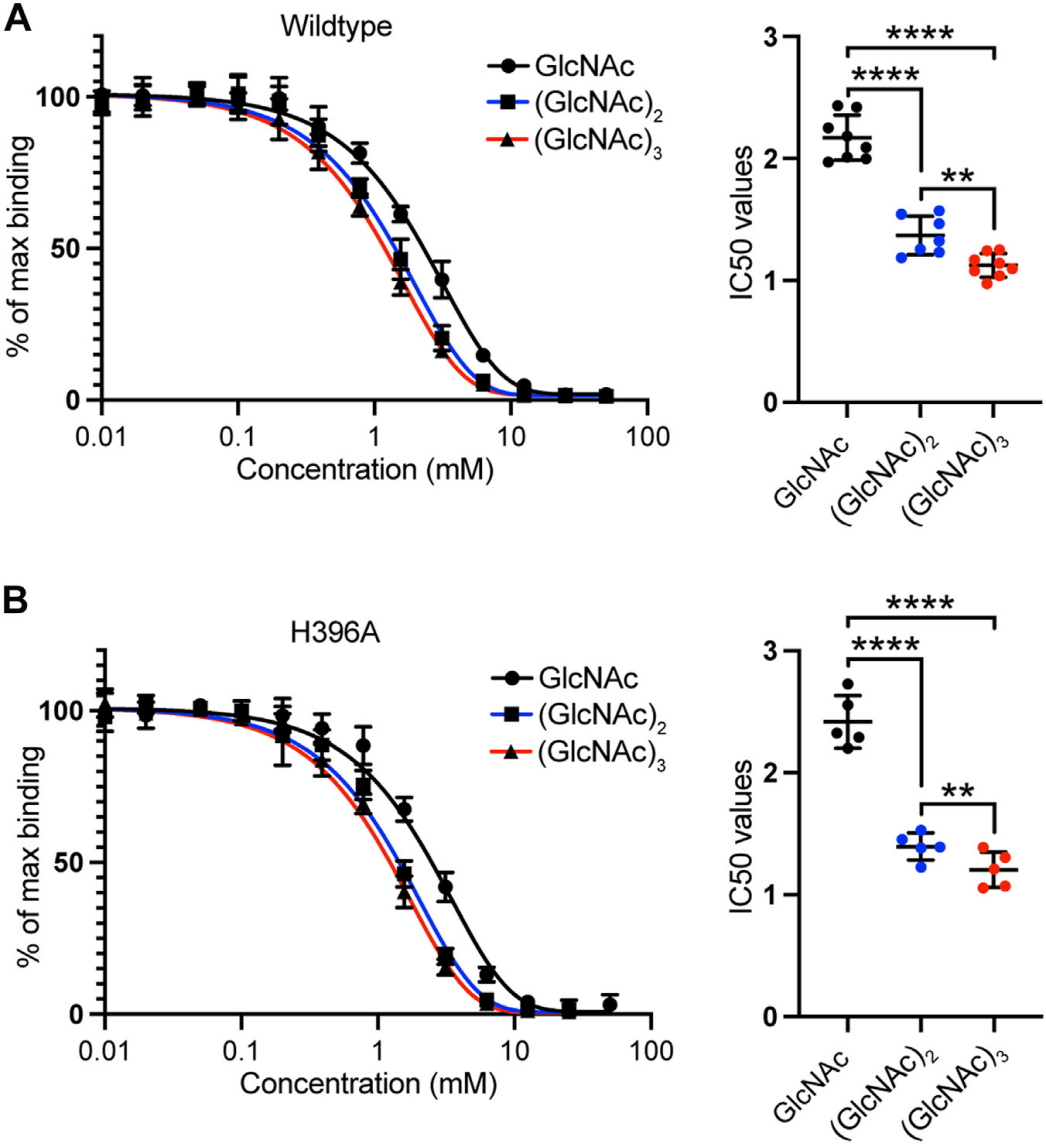
**Repeated measurements of GlcNAc, (GlcNAc)**_**2**_**, and (GlcNAc)**_**3**_**inhibition of FIBCD1-FReD variants binding to acetylated bovine serum albumin.** Repeated measurements demonstrating acetyl group–specific inhibition of binding between (*A*) wildtype (WT) FIBCD1-FReD and (*B*) mutant variant H396A FIBCD1-FReD to immobilized *acetylated bovine serum albumin* by the tested *N*-acetylchitooligosaccharides. Results displayed are combined data from eight and five independently performed experiments, respectively. Statistics: Data are presented as mean ± SD. ∗*p* < 0.05, ∗∗*p* < 0.01, ∗∗∗*p* < 0.001, ∗∗∗∗*p* < 0.0001 by one-way analysis of variance (ANOVA) with *post hoc* Tukey test.

To study the finer details of short oligomer recognition, binding of GlcNAc, (GlcNAc)_2_, and (GlcNAc)_3_ was further investigated by ELISA. Repeated measurements showed that (GlcNAc)_2_ and (GlcNAc)_3_ (IC50 1.4 ± 0.16 mM and 1.1 ± 0.10 mM, respectively) were significantly better at inhibiting the interaction to AcBSA than GlcNAc (IC50 2.2 ± 0.19 mM) (Fig. 6*A*). Moreover, although the difference was small, (GlcNAc)_3_ was found to consistently inhibit the binding significantly better than (GlcNAc)_2_, further suggesting that increasing the number of GlcNAc residues decreases the IC50 values.

To investigate the possible role in the binding of extended ligands of the additional S1-binding site pockets S1(2) and S1(3), as seen in the (fortuitous) native structure, a residue in each of these pockets was mutated and the binding of GlcNAc, (GlcNAc)_2_, and (GlcNAc)_3_ compared. For S1(2), the mutation K381L was chosen to block this site. In the crystal structure of the homologous protein ficolin-3 the overall topology of this pocket is similar but the Leu381 side chain extends into the site and a similar orientation in FIBCD1 would preclude the ligand binding in this pocket (see Fig. S5). The K381L mutation, however, did not bind to the AcBSA used in the ELISA setup (see Fig. S6). This was tested with two different detection antibodies, with different epitopes, suggesting the lack of binding in this assay for K381L may be due to the mutation disrupting the binding to AcBSA rather than due to the mutation inhibiting the detection with the antibodies used. For the S1(3) site, the mutation H396A was used. There is no significant difference in affinity for H396A compared with wildtype for each GlcNAc ligand investigated (see Fig. S6*C*). Similar to wildtype, (GlcNAc)_2_ and (GlcNAc)_3_ (IC50 1.4 ± 0.11 mM and 1.2 ± 0.14 mM, respectively) were significantly better at inhibiting the interaction with the H396A mutant than GlcNAc (2.4 ± 0.22 mM) (see Fig. 6*B*).

## Discussion

The positioning and orientation of the *N*-acetyl group in the subunit A S1(1) pocket is conserved across all the ligand-bound structures, and in the bound N-linked glycan in the native structure, even though there is a 180° rotation about the C–N bond when comparing the pyranose rings of the *N*-acetylated sugars derived from glucose and galactose (Figs. 1 and 2). In all cases the *N*-acetyl interacts with Cys414 N, His415 N, and Tyr431 OH (Table 1). The B factors are lower for the acetyl substituents compared with the saccharides indicating that the *N*-acetyl group, whose binding is highly conserved (5), is more tightly bound, whereas the saccharides have more flexibility in position and orientation. Both the GlcNAc and ManNAc (10) structures reveal a similar disposition of the pyranose ring alongside the conserved interactions of the *N*-acetyl group in the S1(1) pocket, and both are bound by FIBCD1-FReD with comparable affinities (Fig. 5). FIBCD1 thus has the versatility to recognize diverse *N*-acetyl gluco-, galacto- and manno-pyranose monosaccharides underpinned by the core requirement of a conserved mode of *N*-acetyl recognition in S1(1).

Inhibition assays show significant difference between (GlcNAc)_2_ and GlcNAc binding of FIBCD1-FReD (Fig. 6). In contrast, the GlcNAc and (GlcNAc)_2_ bound structures both show a single GlcNAc in the S1-binding site S1(1) pocket with closely similar structures, although the GlcNAc in the (GlcNAc)_2_ bound structure is rotated/tilted slightly in the direction of the S1(2) site resulting in an additional contact with the protein at Tyr405 (see Fig. 2*A*). Neu5Ac also has an additional interaction with Tyr405 (see Table 1), and this too has a lower IC50 than GlcNAc (1).

**Figure 7:**
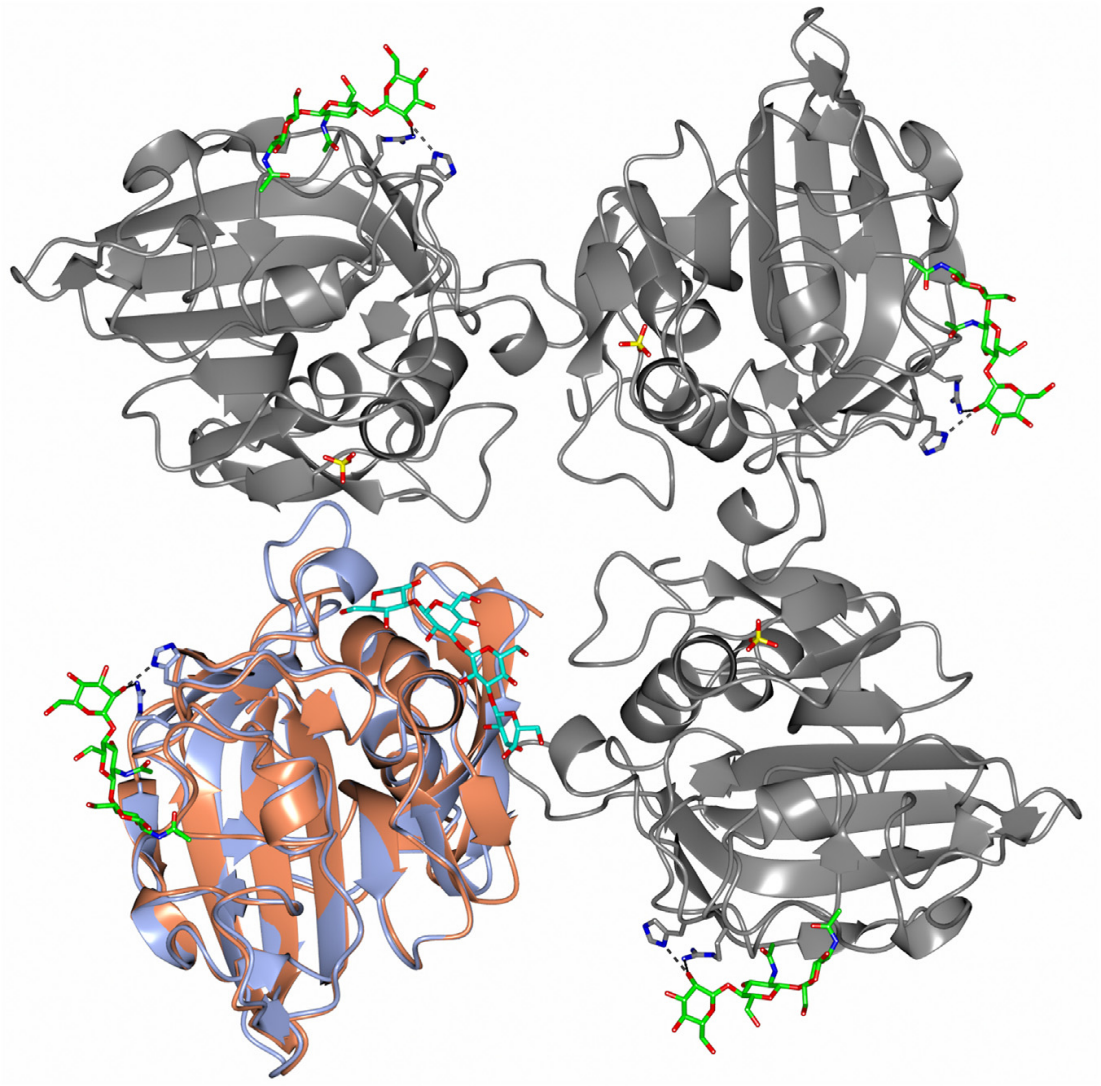
**Quaternary structure of FIBCD1-FReD.** The subunit B–derived tetramer from the native structure with bound glycan (*green*) and the interacting His396 and Arg412 side chains in the S1(3) pocket are shown. A subunit from the ficolin-2 fungal 1,3-β-D-glucan bound structure (*orange*) with ligand (*cyan*) connecting the S3 and S4 binding sites is overlaid (least-squares fit with main chain equivalent residues in PDB ID 2J0Y subunit C) onto one of the FIBCD1 subunits (*blue*). The sulfate ion located in the S3 site in FIBCD1 is shown in the other three subunits.

Binding studies also show a significant increase in affinity for (GlcNAc)_3_ compared with GlcNAc, and there is also a clear binding preference for longer *N*-acetylchitooligosaccharides ((GlcNAc)_11_ > (GlcNAc)_5_ > (GlcNAc)_3_ > GlcNAc), suggesting that longer chains interact with FIBCD1-FReD across an extended binding surface. How this is achieved may be similar to that seen in the CHO-derived native structure where, fortuitously, the N-linked glycosylation from subunit A is bound by subunit B in an extended S1-binding site on the FIBCD1-FReD surface (Figs. 3 and 7). The first GlcNAc residue in the glycosylation chain interacts with the subunit B ligand-binding site S1(1) in essentially the same manner as the other bound ligands; the second GlcNAc crosses the Cys414–His415 backbone allowing the *N*-acetyl group to fit into a pocket delineated by the residues His382 and Arg412–His415, establishing a second acetyl-binding pocket S1(2). Mannose, the third carbohydrate of the CHO-derived glycan in the native structure, further extends the glycan-binding site to a third pocket S1(3) by interacting with the His396 and Arg412 side chains. Although there is a lack of sequence and structural homology with FIBCD1, chitin-binding hevein-like proteins exhibit a three-pocket chitin-binding site, which extends from the primary acetyl-binding site, but in the opposite direction to FIBCD1, from nonreducing to reducing terminal (19). Inhibition binding data on the FIBCD1 H396A mutant show no significant difference to wildtype, indicating that the S1(3) site is not critical in binding (GlcNAc)_3_. It was not possible to assess the effect of the binding of GlcNAc ligands to the K381L mutant in the S1(2) site for the ELISA setup used here. Two different detection antibodies, with different epitopes, were tested, suggesting that this mutant does not bind AcBSA and that S1(2) could be important in binding acetylated ligands. It is not clear why K381L does not appear to bind AcBSA as purification of the mutants based on their ability to bind acetylated Toyopearl resin indicates that this FIBCD1 mutant is able to bind acetylated ligands. It may be that either the S1(2)-binding pocket plays a major role in AcBSA, but not Toyopearl, binding or the S1(1) site is disrupted alongside the S1(2) mutation and Toyopearl, but not AcBSA, utilizes the additional binding pockets.

While crystal structures of other FReD-containing proteins, including ficolin-2, have revealed the presence of functional binding surfaces across which extended ligands may bind (11), for example, ficolin-2 binds 1,3-β-D-glucan, a molecular marker of fungal infection, across a 25-Å binding surface at the S3 and S4 binding sites (Fig. 7), an extended surface at S1 has not previously been identified. The *N*-acetyl S1(1) pocket is conserved in FIBCD1, ficolins, and TL5A (11, 14); however, aligning the FIBCD1-FReD S1(2) and S1(3) binding pockets with the homologous ficolins and TL5A reveals a lack of sequence conservation (Fig. 3*B*), although it may be possible structurally that ficolin-1 could bind a second GlcNAc in a similar manner to FIBCD1. In general, while His382, Cys414, and His415 in the secondary acetyl-binding pocket S1(2) are largely conserved in TL5A and ficolins, the residues corresponding to Arg412 and Asn413 in S1(2) are not. This suggests that any extended S1 site in TL5A and ficolins may exhibit different recognition properties.

Sulfate ions are also present in the S1(2) and S1(3) binding pockets, in subunit A of the native structure and subunit B of the ligand-bound structures, respectively. Each of these sulfate ions interacts with the same residues as the S1(2) GlcNAc and S1(3) mannose of the subunit A glycan bound by subunit B in the native structure (Table 1). There is also electron density in the S1(3) pocket in several of the structures, suggesting the presence of acetate coordinated by His396 and Arg412, although these cannot be modeled with confidence. The S1(1) pocket, however, displays a clear preference for acetylated ligands with the GalNAc-4S ligand bound *via* the *N*-acetyl group and acetate ions occupying this pocket in subunit A of the native structure and subunit B of the GlcNAc, GalNAc-4S, Neu5Ac, and (GlcNAc)_2_-bound structures. FIBCD1 has been shown to bind to proteoglycans (6), which the structures here suggest may be accomplished *via* a multiple-pocket S1 site.

The glycan, acetate, and sulfate interactions seen in the structures in the S1(1), S1(2), and S1(3) pockets (Fig. 3*C*) suggest that longer ligands may be bound by FIBCD1 *via* an extended surface whose direction of extension, from S1(1) across S1(2), is implied by the similarity in orientation of the bound S1(1) GlcNAc residue from the native glycosylation and in the GlcNAc and (GlcNAc)_2_ ligand-bound structures. Thus, further GlcNAc carbohydrates in an oligomer may extend in this direction, with the glycosylation from the neighboring protein in the S1(2) site indicating how recognition can be achieved. While it has not been possible to definitively establish that the S1(2) pocket is used to bind acetylchitooligosaccharides, the combined evidence suggests a putative role for this site and a functional binding surface across which FIBCD1 may coordinate longer *N*-acetylchitooligosaccharides and other markers of infection (Figs. 3 and 7). Whether longer chitin fragments are able to cross-link different subunits, as suggested for hevein (17), or different binding sites, as seen for ficolin-2 (11), in the same or neighboring tetramers remains to be seen. What is clear is that FIBCD1 has the ability to coordinate a broad range of ligands suggesting that, similar to the ficolins, FIBCD1-FReD has broad ligand specificity. While the S1(1) pocket shows a clear, strong preference for *N*-acetyl binding, the structures presented here reveal a versatility in recognition of *N*-acetyl, sulfate, or other carbohydrates in S1(2) suggesting the ability to recognize a diverse range of ligands across an extended S1 binding surface. Investigation of FIBCD1-FReD with chitin, GAGs, longer *N*-acetylchitooligosaccharides, other pathogen-associated carbohydrate and other ligands, together with examination of potential effector mechanisms, will provide further insight and understanding into the structure, function, and recognition properties of FIBCD1.

## Experimental procedures

### Cloning, expression, and purification of FIBCD1-FReD

The cloning, expression, and purification of FIBCD1-FReD have been described in detail (1). Briefly, for the protein used for the ligand-bound crystal structures, the initial enzyme-linked immunosorbent inhibition assay (ELISA) and the MST affinity assay, FIBCD1-FReD (residues 236–461) was expressed in Sf9 insect cells using the pNT-Bac vector expression system. The recombinant FIBCD1-FReD variants used for the native crystal structure and for the repeated measurements of (GlcNAc)_n_ n = 1, 2, 3 inhibition by ELISA, the same fragments of FIBCD1-FReD (wildtype, H396A, and K381L) were expressed in Chinese hamster ovary (CHO) cells using the ExpiCHO expression system (ThermoFisher Scientific) with FIBCD1-FReD (including the IgG K signal peptide for secretion) cloned into the pcDNA3.1+ plasmid (Genscript). Purification of the FIBCD1-FReD variants, regardless of the expression system, was achieved through affinity chromatography using acetylated Toyopearl AF-Amino-650M resin (Tosoh) followed by ion-exchange chromatography using a Resource Q ion-exchange column (GE Healthcare) as outlined in Schlosser *et al.* (Ref (1)).

### ELISA inhibition assays

The specificity of the binding of recombinant FIBCD1-FReD, expressed in insect cells, to AcBSA was assayed in technical duplicates by inhibition with acetylated compounds, including GlcNAc (Sigma-Aldrich) and three *N*-acetylchitooligosaccharides of defined length ((GlcNAc)_3_, (GlcNAc)_5_, and (GlcNAc)_11_) and acetylation (66%), which were kindly provided by Professor Morten Sølie (Norwegian University of Life Sciences). The monoclonal antibodies used in the study here were generated in-house at the University of Southern Denmark and chosen from a large series of monoclonal antibodies raised against the FReD domain (human) in FIBCD1-deficient mice—details for the production are given in (3). Microtiter plates (MaxiSorp) were coated with 1 μg/ml AcBSA (Sigma-Aldrich) or bovine serum albumin (Sigma-Aldrich) and blocked with 10 mM Tris, 140 mM NaCl, pH 7.5 containing 0.05% Tween-20 (TBS/Tw) and 5 mM CaCl_2_. Using a constant concentration of recombinant FIBCD1-FReD (50 ng/ml), samples of FIBCD1-FReD were mixed with inhibitors to give final inhibitor concentrations of 0 to 50 mM in TBS/Tw and 5 mM CaCl_2_. The mixtures were incubated overnight in the AcBSA-coated wells at 4 °C. After overnight incubation at 4 °C and washing with TBS/Tw and 5 mM CaCl_2_, the wells were incubated for 2 h at room temperature with 1 μg/ml biotinylated monoclonal anti-FIBCD1-FReD antibody (clone 11-14-25) in TBS/Tw and 5 mM CaCl_2_. The plates were washed with TBS/Tw containing 5 mM CaCl_2_ and incubated for 1 h with HRP-conjugated streptavidin (ThermoFisher) diluted in 1:2000 in TBS/Tw containing 5 mM CaCl_2_ followed by washing and developing with TMB substrate according to the manufacturer’s instructions (ThermoFisher). Additional ELISA-based inhibition studies, utilizing the mammalian expressed recombinant FIBCD1-FReD (wildtype and H396A variants), were conducted in technical duplicates following the same protocol as outlined above with the inhibitory compounds GlcNAc, (GlcNAc)_2_, and (GlcNAc)_3_ purchased from Megazyme. To validate the inability of FIBCD1-FReD mutant variant K381L to bind AcBSA in the ELISA-based setups, the monoclonal anti-FIBCD1-FReD antibody (clone 11-14-25) used throughout this study was compared with results using the monoclonal anti-FIBCD1-FReD antibody (clone 11-14-13) with a different epitope.

### Microscale thermophoresis affinity assays

The affinity between recombinant FIBCD1-FReD and the acetylated sugars GlcNAc, (GlcNAc)_5_ and ManNAc as well as AcBSA was determined by MST. FIBCD1-FReD expressed by insect cells was labeled using the Monolith NT Protein Labeling Kit RED-NHS (NanoTemper Technologies) according to the manufacturer’s instructions. Removal of unreacted dye was performed with the supplied columns equilibrated in TBS containing 5 mM CaCl_2_. Labeled FIBCD1-FReD was diluted in assay buffer (TBS/Tw containing 5 mM CaCl_2_) and used at a constant concentration of 30 nM throughout the experiments, whereas the putative ligands were titrated in 2-fold dilutions, ranging from 0.6 nM to 10 μM (AcBSA), 100 μM to 200 mM (GlcNAc), 50 μM to 100 mM (GlcNAc)_5_, and 250 μM to 500 mM (ManNAc). For the measurements, all ligands were incubated with labeled FIBCD1-FReD for 20 min at RT (21–22 °C) before being loaded into standard capillaries (NanoTemper Technologies). Measurements were performed using a Monolith NT.115 instrument (NanoTemper Technologies) at an ambient temperature of 25 °C. Instrument parameters were adjusted to 40% LED power, 80% MST power, and a laser-on time of 30 s and a laser-off time of 5 s. Data from three to four independently pipetted experiments were analyzed using the NanoTemper Analysis 1.2.20 software and used to fit the data for determination of the *K*_d_ values.

### Crystallization and data collection

Crystals of the fibrinogen domain (residues 236–461) were grown in sitting drops consisting of an equal volume (1.5–2 μl) of protein solution and precipitant solution (7–9% dioxane, 0.1 M Mes pH 6.5, and 1.5–1.7 M (NH_4_)_2_SO_4_). Native crystals of FReD were grown using protein expressed in Sf9 insect cells, and ligands were introduced into the crystals by the addition of ligands to the cryobuffer. Successive additions of 2- to 3-μl aliquots of increasing concentrations (5–25%) of glycerol cryobuffer were made to each well, followed by the addition of further 2-μl aliquots of 25% glycerol cryobuffer and an exchange of ∼10 μl of the microbridge solution with 25% glycerol cryobuffer. The concentration of each ligand (all purchased from Sigma-Aldrich) in the respective cryobuffer was 100 mM *N*-acetylalanine, 100 mM GlcNAc, 50 mM *N*-acetylgalactosamine-4-sulfate (GalNAc-4S), 125 mM *N*-acetylneuraminic acid (Neu5Ac), and 100 mM *N*,*N*′-diacetylchitobiose ((GlcNAc)_2_). For the GalNAc-4S structure, crystals were initially soaked with *N*-acetylgalactosamine (GalNAc), as per the protocol above, before undergoing a second exchange of ∼10 μl cryobuffer containing GalNAc-4S at the listed concentration. For the native structure, native crystals of FReD were grown as outlined above using the CHO expressed protein. A mixed chondroitin sulfate A/C (CSA/C) oligomer (Iduron) was introduced into crystals by the addition of three 0.75-μl aliquots of 100 mM CSA/C to the microbridge. This was followed by the addition of three 0.5-μl aliquots of 70% glycerol to the microbridge for cryoprotection prior to data collection. Following data processing, no ligand was found to be present in the crystal structure.

Data were collected at Diamond Light Source, from a single crystal in each case, using an ADSC Quantum 315 (Neu5Ac and (GlcNAc)_2_ datasets) and Pilatus 6M-F (Native, *N*-acetylalanine, GlcNAc, and GalNAc-4S datasets) detector. Integrated intensities were calculated using *MOSFLM* (22) and data were processed using the *AIMLESS*, *TRUNCATE*, *UNIQUIFY*, and *SORTMTZ* programs as part of the CCP4 program suite (23). Data collection and processing statistics are given in Table 2.

**Table 2:** Crystallographic data and refinement statistics for native and ligand-bound FIBCD1

	Native		AlaNAc bound		GlcNAc bound		GalNAc-4S bound		Neu5Ac bound		(GlcNAc)_2_ bound	
Data collection												
Synchrotron station	DLS I03		DLS I04		DLS I02		DLS I04-1		DLS I04		DLS I04	
Wavelength (Å)	0.9762		0.9795		0.9795		0.9200		0.9702		0.9795	
Space group	P4		P4		P4		P4		P4		P4	
Cell dimensions (Å)	a=b=118.35 c=44.09 α=β=γ=90º		a=b=119.22 c=44.18 α=β=γ=90°		a=b=113.60 c=44.08 α=β=γ=90°		a=b=119.02 c=44.31 α=β=γ=90°		a=b=119.29 c=44.21 α=β=γ=90°		a=b=118.67 c=44.23 α=β=γ=90°	
Resolution range (Å)	83.69–2.00 (2.05–2.00)		84.30–1.94 (1.99–1.94)		56.80–1.93 (1.98–1.93)		53.23–1.97 (2.02–1.97)		59.64–1.85 (1.89–1.85)		59.33–1.84 (1.88–1.84)	
Observations	152,224 (10,903)		145,767 (9799)		147,738 (8913)		168,534 (9650)		183,426 (9655)		150,660 (9208)	
Unique reflections	41,341 (3000)		46,069 (3105)		42,095 (2759)		42,961 (3088)		52,803 (3200)		53,600 (3248)	
Completeness (%)	99.1 (99.3)		99.1 (98.8)		98.7 (98.5)		97.3 (98.1)		98.6 (98.7)		99.5 (99.7)	
R_merge_^a^	0.103 (0.343)		0.072 (0.358)		0.063 (0.342)		0.097 (0.245)		0.066 (0.345)		0.059 (0.299)	
CC1/2	0.992 (0.846)		0.995 (0.770)		0.997 (0.796)		0.976 (0.888)		0.993 (0.795)		0.997 (0.861)	
Mean (I/σ(I))	8.3 (3.7)		9.4 (3.0)		12.4 (3.0)		7.5 (3.0)		11.7 (3.0)		9.8 (2.9)	
Refinement												
Protein atoms	3520		3508		3520		3514		3520		3520	
Residues (chain A)	239–457		240–457		239–457		239–457		239–457		239–457	
Residues (chain B)	239–457		240–457		239–457		240–457		239–457		239–457	
Glycan on subunit A	NAG-NAG-MAN		NAG-FUC		NAG-(FUC,FUC) -NAG		NAG-FUC		NAG-(FUC)-NAG		NAG-FUC	
Water molecules	234		248		248		235		350		337	
Other molecules												
Subunit	A	B	A	B	A	B	A	B	A	B	A	B
Calcium ion	1	1	1	1	1	1	1	1	1	1	1	1
Ligand	-	-	1	1	1	-	1	-	1	-	1	-
Sulfate ion	2	1	1	2	2	2	1	2	1	2	1	2
Acetate	2	1	2	1	1	2	-	1	1	2	-	3
Glycerol	-	1	1	-	-	-	1	-	-	-	1	-
R_work_ (%)^b^	0.166		0.189		0.167		0.184		0.175		0.161	
R_free_ (%)^c^	0.184		0.210		0.191		0.226		0.196		0.176	
Bond length r.m.s.d (Å)	0.0044		0.0018		0.0068		0.0048		0.0038		0.0073	
Bond angles r.m.s.d (°)	1.304		1.178		1.409		1.351		1.2605		1.4312	
Average B-values (Å^2^)												
Protein	16.96		22.82		18.87		24.63		17.13		21.30	
Water	27.38		33.59		31.21		34.75		30.62		34.72	
Ligands	41.25		48.52		47.17		66.27		38.22		49.29	
PDB ID	6ZR4		6ZR0		6ZQR		6ZR3		6ZQY		6ZQX	
Ramachandran plot values (%)^d^												
Favored	95.39		95.83		95.39		95.84		96.08		95.85	
Outliers	0.00		0.0		0.0		0.0		0.0		0.0	

Values in parentheses correspond to the highest resolution bin.

a R_merge_ = Σ_h_Σ_j_ | I_h,j_ – I_h_ |/Σ_h_Σ_j_ | I_h,j_|, where I_h,j_ is the j^th^ observation of reflection *h* and I_h_ is the mean of the *j* measurements of reflection *h*.

b R_work_ = Σ_h_ | |F_oh_| - |F_ch_| |/Σ_h_ |F_oh_|, where F_oh_ and F_ch_ are the observed and calculated structure factor amplitudes, respectively, for reflection *h*.

c R_free_ is equivalent to R_work_ for a randomly selected subset (5%) of reflections not used in the refinement.

d Determined according to MolProbity.

### Structure solution and refinement

Isomorphism was sufficient to allow the atomic coordinates of the previously determined 2.0 Å native structure of FIBCD1-FReD (PDB code: 4M7H; (10)) to be used as the starting model for the FIBCD1 structures except for the GlcNAc ligand-bound structure. For this latter dataset, which has a slightly smaller tetragonal unit cell, molecular replacement using *MOLREP* was used; this gave a solution that placed the two independent subunits in approximately the same location and orientation as all the other structures suggesting that the starting model was too far removed from the solution as an initial starting point. Initial models were built in Coot (24) after rigid body refinement with *REFMAC5* (25) and subsequent model building was completed over multiple rounds of restrained refinements using *REFMAC5* alternated with rounds of manual model building with Coot. Ligand coordinates were imported *via* Coot from PDB. The quality of the final structures was verified using the MolProbity server and *PRIVATEER* as part of the CCP4i2 suite (26, 27) and the PDB validation software. Final refinement statistics are provided in Table 2. Molecular figures were generated using CCP4mg (28). Multiple sequence alignment was generated using Clustal Omega (29) using sequence information from the Swiss-Prot UniProtKB database (30) and structural data from the PDB.

## Data availability

The coordinates and structure factors for the native (6ZR4) and ligand-bound *N*-acetylalanine (6ZR0), GlcNAc (6ZQR), GalNAc-4S (6ZR3), Neu5Ac (6ZQY), and (GlcNAc)_2_ (6ZQX) structures have been deposited with and are freely available from the PDB. Diffraction data are available *via* Keele University Data Repository (https://doi.org/10.21252/2ndt-aq43; https://doi.org/10.21252/r2nx-0425; https://doi.org/10.21252/2b3f-9369; https://doi.org/10.21252/hx7e-rd04; https://doi.org/10.21252/403p-sz47; https://doi.org/10.21252/zcfy-cw20). All remaining data are contained within the article.

## Supporting information

This article contains supporting information (11).

## Conflict of interest

The authors declare that they have no conflicts of interest with the contents of this article.
