# Supplementary material for: Crystal structures of human immune protein FIBCD1 suggest an extended binding site compatible with recognition of pathogen-associated carbohydrate motifs
Source: J Biol Chem. 2023 Dec 10;300(1):105552. doi: 10.1016/j.jbc.2023.105552 (PMC10825690; doi:10.1016/j.jbc.2023.105552)
Supplement: Supporting information [file mmc1.pdf]

Crystal structures of human immune protein FIBCD1 suggest an extended binding site compatible with recognition of pathogen associated carbohydrate motifs

Harry M. Williams[1], Jesper B. Moeller[2,3], Ian Burns[1], Anders Schlosser[2], Grith L. Sorensen[2], Trevor J. Greenhough[1], Uffe Holmskov[2] and Annette K. Shrive[1*]

Figure S1
Figure S2
Figure S3
Figure S4
Figure S5
Figure S6

Figure S1

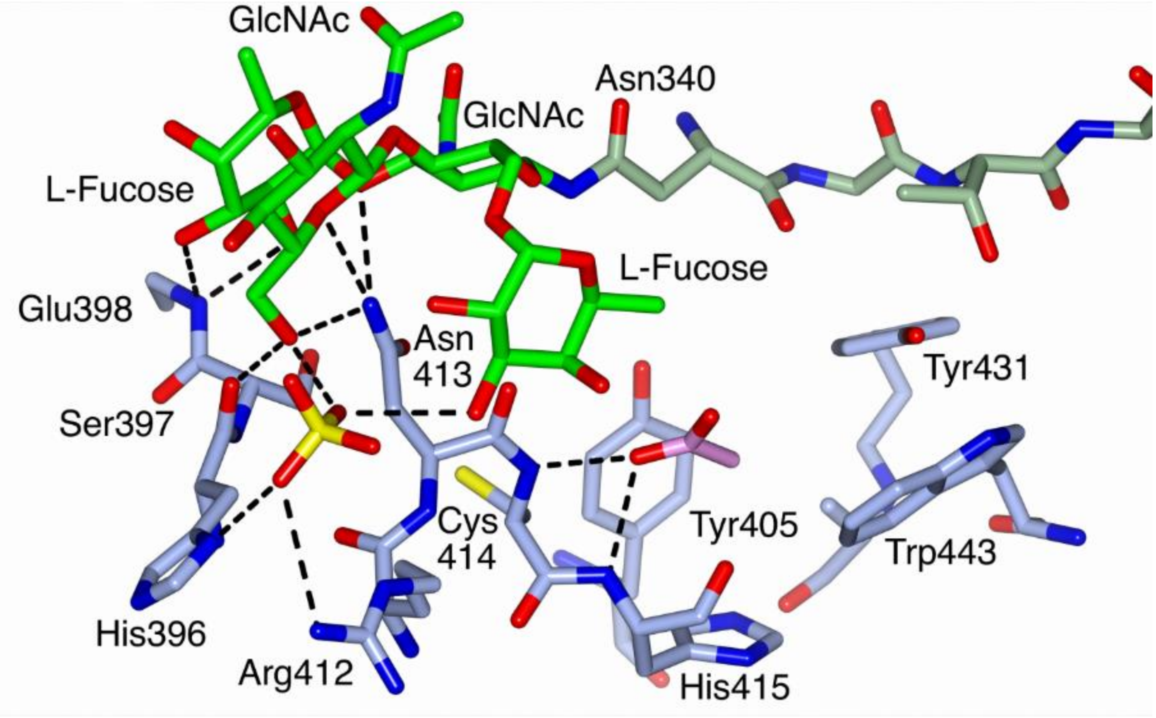

Figure S1. Glycan interactions in the ligand bound structures. The Asn340 N-linked glycan (green) in subunit B (blue) of the GlcNAc bound structure. Acetate (pink) in S1(1) and sulfate (yellow) in S1(3) are shown along with protein and glycan interactions.

Figure S2

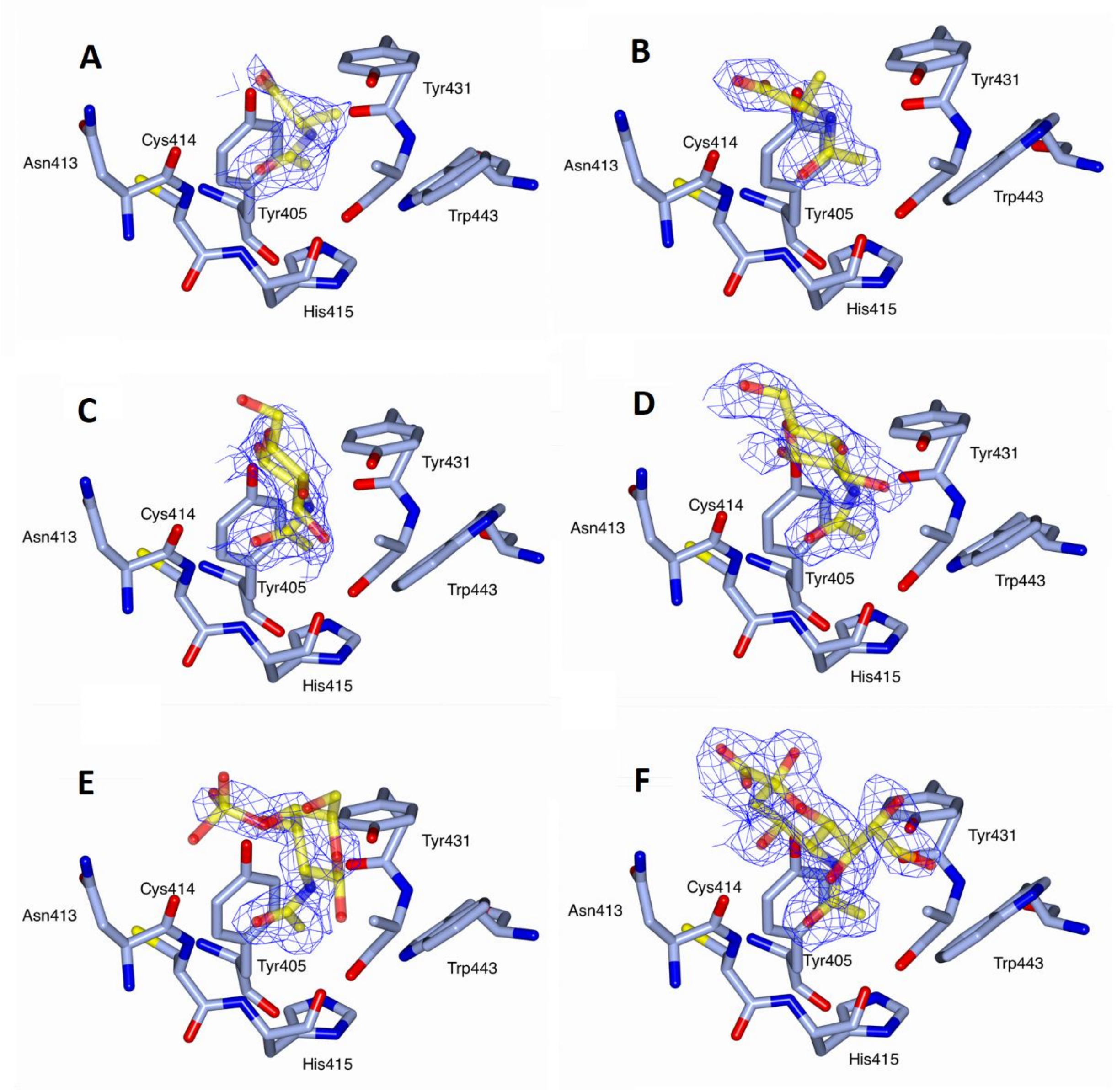

Figure S2. Electron density omit maps for each ligand. Ligands (yellow) bound to the S1(1) ligand-binding site (pale blue) with key amino acid residues labelled. (**A**) N-acetylalanine (subunit A); (**B**) N-acetylalanine (subunit B); (**C**) GlcNAc; (**D**) $(GlcNAc)_2$ (only one GlcNAc residue fitted); (**E**) GalNAc-4S; (**F**) Neu5Ac. Omit maps were produced by deleting the fitted ligands from each PDB and re-refining the protein structure in Refmac5. Ligands were then superposed for visualization purposes. Electron density at 50% transparency, clipped around the ligand coordinates, is shown in purple at the 0.7σ level. Images produced using CCP4mg.

Figure S3

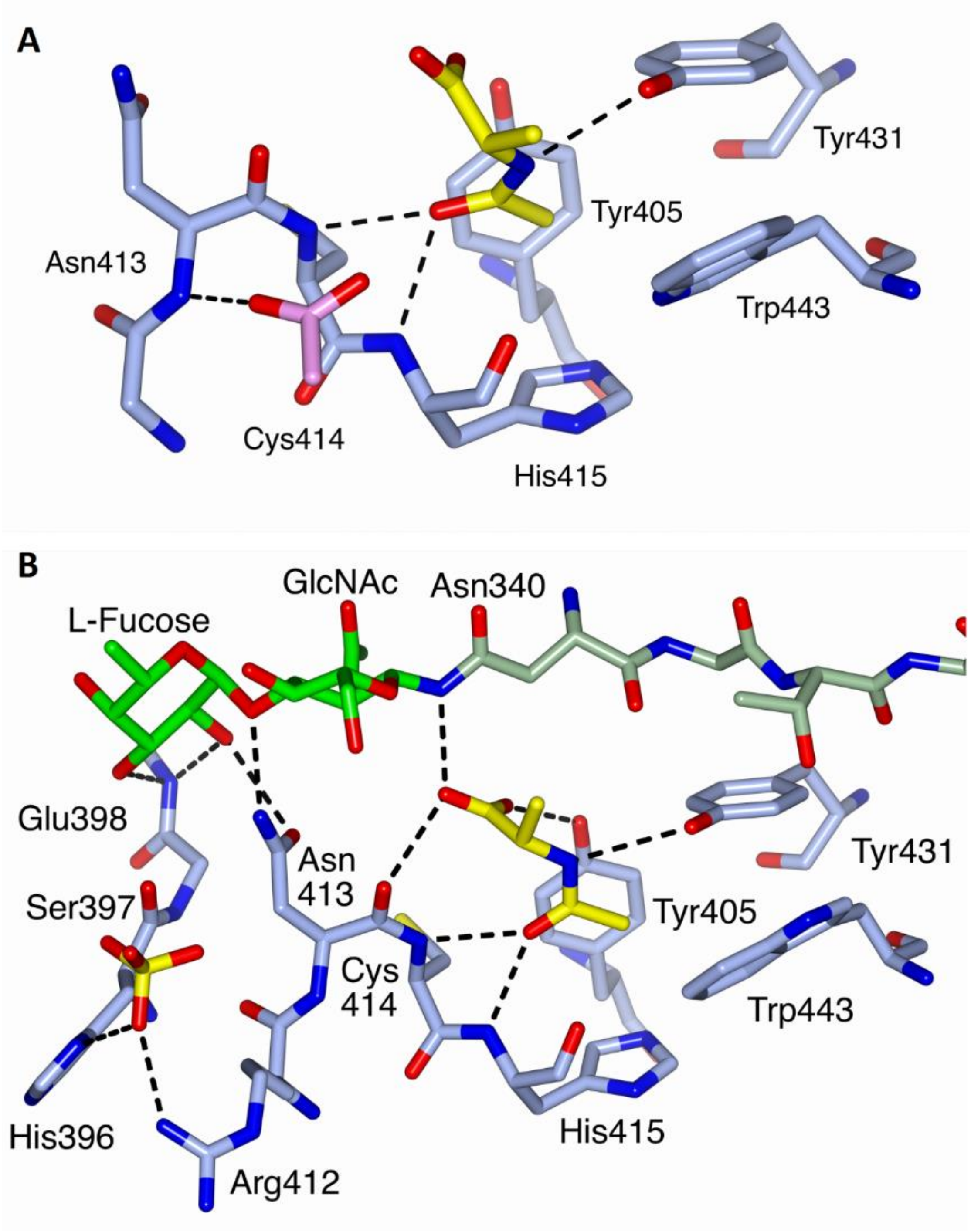

Figure S3. N-acetylalanine binding in FIBCD1. Bound N-acetylalanine ligand (yellow) shown in the S1(1) ligand-binding pocket. Interactions of the ligand with protein (blue) are shown by dashed lines. (**A**) Subunit A showing the acetate ion (pink) located in the neighbouring S1(2) pocket. (**B**) Subunit B showing the sulfate ion in the S1(3) pocket and the subunit A N-linked glycan.

Figure S4

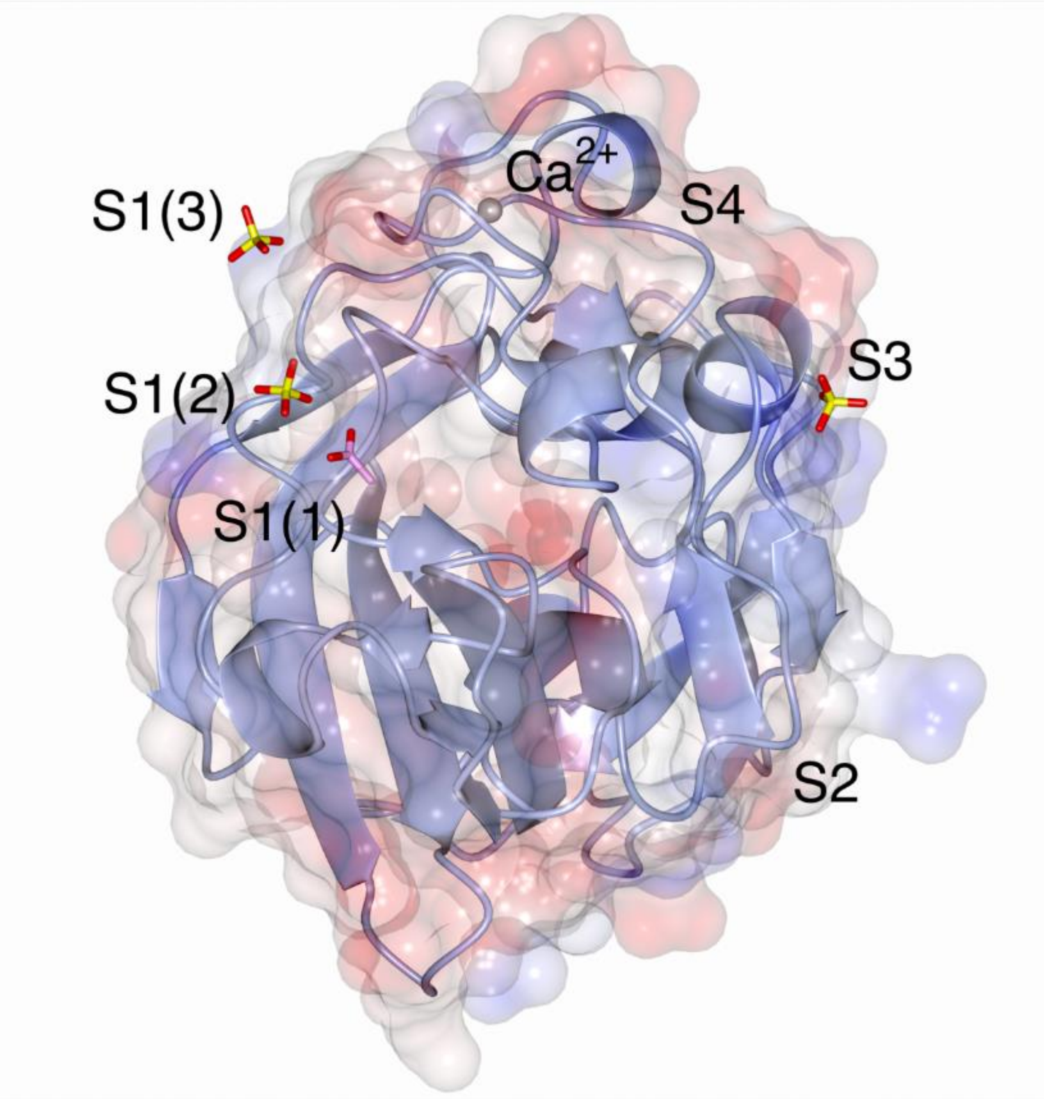

Figure S4. FIBCD1-FReD subunit with S1-S4 sites highlighted. Subunit of FIBCD1 FReD with the extended S1 site and ficolin-associated S2, S3 and S4 sites labelled (see reference 11). For clarity the acetate (pink) and sulfate (yellow) ions located at these sites in FIBCD1 are shown as sticks along with the calcium ion as a grey sphere. As described in the text, across the various structures and subunits the S1(1) pocket is occupied by acetate or by ligand/glycan N-acetyl, the S1(2) pocket when occupied contains glycan N-acetyl, sulfate or acetate, while the S1(3) pocket is occupied by glycan mannose or sulfate in all B subunits but is empty in all A subunits.

Figure S5

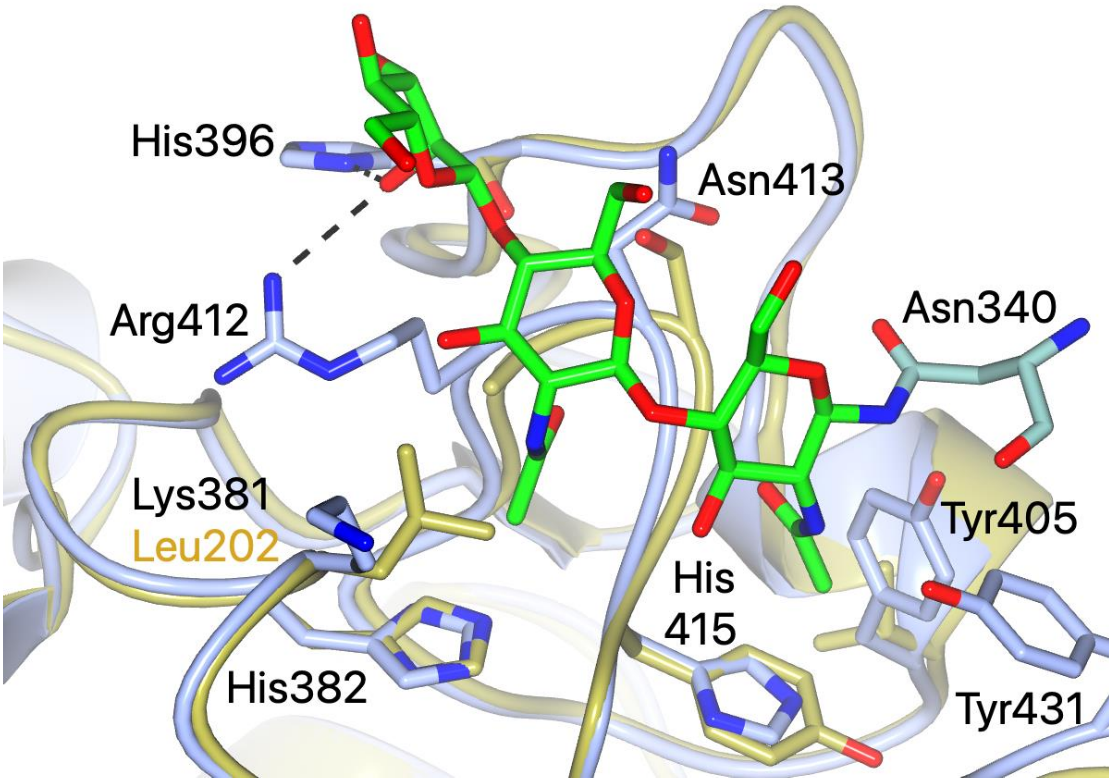

Figure S5. Comparison of the extended ligand binding site in FIBCD1 with ficolin-3. Overlay of FIBCD1 subunit (pale blue) and ficolin-3 subunit (gold) generated by a least-squares fit of main chain atoms in FIBCD1 with equivalent residues in PDB 2J60. The FIBCD1 native Asn340-linked glycan is shown in green bound across the extended FIBCD1 ligand binding site with selected sidechains shown, including the FIBCD1 residues mutated for binding studies, His396 and Lys381 (with the ficolin-3 equivalent residue Leu202).

Figure S6

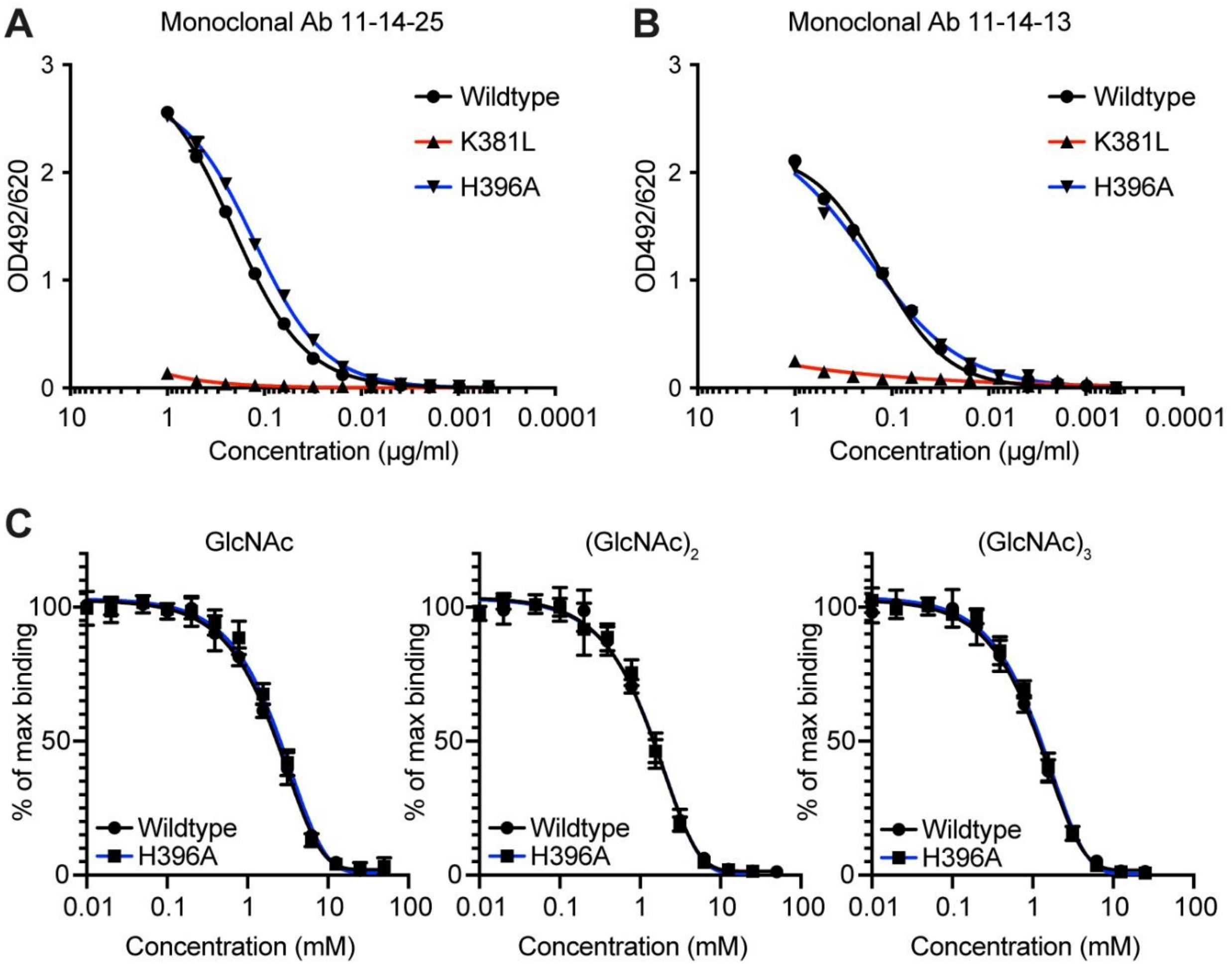

Figure S6. Binding characteristics of wildtype and mutant FIBCD1-FReD variants. (A and B) ELISA-based setups demonstrating binding of wildtype and mutation H396A but not K381L to acetylated BSA using 2 different monoclonal antibodies for detection. **(C)** Direct comparison of wildtype and mutation H396A binding to acetylated BSA in the presence of increasing concentrations of the chitooligomers; GlcNAc, $(GlcNAc)_2$ and $(GlcNAc)_3$. Results in A and B are representative of 2 independent experiments (illustrated as mean from technical duplicates), while results in C are combined data from 5-8 independent experiments, presented as mean ± SD.
